# Neutrophils in Tumorigenesis: Missing Targets for Successful Next Generation Cancer Therapies?

**DOI:** 10.3390/ijms22136744

**Published:** 2021-06-23

**Authors:** Fabrice Tolle, Viktor Umansky, Jochen Utikal, Stephanie Kreis, Sabrina Bréchard

**Affiliations:** 1 Department of Life Sciences and Medicine, University of Luxembourg, L-4367 Belvaux, Luxembourg; fabrice.tolle@uni.lu (F.T.); sabrina.brechard@uni.lu (S.B.); 2 Skin Cancer Unit, German Cancer Research Center (DKFZ), 69121 Heidelberg, Germany; v.umansky@dkfz.de (V.U.); jochen.utikal@umm.de (J.U.); 3 Department of Dermatology, Venereology and Allergology, University Medical Center Mannheim, Ruprecht-Karl University of Heidelberg, 68167 Mannheim, Germany

**Keywords:** neutrophils, metastasis, immunotherapy, tumorigenesis

## Abstract

Neutrophils—once considered as simple killers of pathogens and unexciting for cancer research—are now acknowledged for their role in the process of tumorigenesis. Neutrophils are recruited to the tumor microenvironment where they turn into tumor-associated neutrophils (TANs), and are able to initiate and promote tumor progression and metastasis. Conversely, anti-tumorigenic properties of neutrophils have been documented, highlighting the versatile nature and high pleiotropic plasticity of these polymorphonuclear leukocytes (PMN-L). Here, we dissect the ambivalent roles of TANs in cancer and focus on selected functional aspects that could be therapeutic targets. Indeed, the critical point of targeting TAN functions lies in the fact that an immunosuppressive state could be induced, resulting in unwanted side effects. A deeper knowledge of the mechanisms linked to diverse TAN functions in different cancer types is necessary to define appropriate therapeutic strategies that are able to induce and maintain an anti-tumor microenvironment.

## 1. Introduction

Immune cells within the tumor microenvironment (TME) interact with tumor cells and play an important role in the process of tumorigenesis and progression of cancer. Tumor-associated macrophages have received much interest in recent years [1,2,3,4,5], while tumor-associated neutrophils (TANs) have been studied much less. Attention is now paid to TANs and their role in the development of cancer and metastasis. Neutrophils, despite their short lifespan [6,7], and through their ability to secrete de novo inflammatory mediators [8,9], are not only involved in the killing of pathogens, but accumulating evidence suggests that they also contribute to the pathogenesis of complex diseases, such as cancer.

Until recently, neutrophils were believed to have their main function in the framework of defense against extracellular pathogens through the process of phagocytosis, release of a large arsenal of effector molecules, production of reactive oxygen species, and the generation of extracellular traps. However, it is clear that neutrophils are multi-functional cells whose disruption or dysregulation can contribute to tumorigenesis.

Besides their functional properties, neutrophils associated with lymphocytes are also considered as biomarkers for cancer prognosis. Indeed, an elevated peripheral blood neutrophil-to-lymphocyte ratio has been reported as a prognostic marker in several cancers and is associated with a poorer overall outcome and progression-free survival in cancer patients [10,11,12,13].

Based on the classification of tumor-associated macrophages, a similar nomenclature has been given to neutrophils with tumor-suppressing (TAN1) and tumor-promoting (TAN2) properties. Fridlender et al. [14], in 2009, postulated that their polarization toward a pro- or anti-tumor state is strictly dependent on the local environmental conditions. Despite many unresolved issues, the phenotypic differences between TAN1 and TAN2 have been comprehensively reviewed before [15,16,17,18,19,20], Table 1, and their main functional differences are briefly summarized in Table 1.

One important point to keep in mind is that studies on TAN polarization into N1 or N2 states have exclusively been carried out in mouse models and, to date, a pro-tumoral neutrophil activity in human tumor tissues has been challenging to demonstrate and characterize [35,36].

## 2. The Exodus of Neutrophils towards the Tumor Microenvironment

Neutrophils are actively recruited to the site of a tumor attracted by conditions (such as necrosis) that signal tissue injury to the TME. In solid tumors, this type of cell death is accelerated by hypoxia, nutrient starvation, or adverse effects induced by immune-checkpoint inhibitors. Necrosis leads to the release of damage-associated molecular pattern molecules (DAMPs), which can recruit and activate neutrophils [37,38]. These neutrophils stimulated by immunogenic cell death acquire pro-inflammatory properties with a cytotoxic effect on tumor cells.

Moreover, neutrophils are attracted, in a temporal and spatial manner, by a set of bioactive lipids (e.g., eicosanoid leukotriene B4), chemokines secreted by tumor and immune cells already present in the TME.

**Figure 1:**
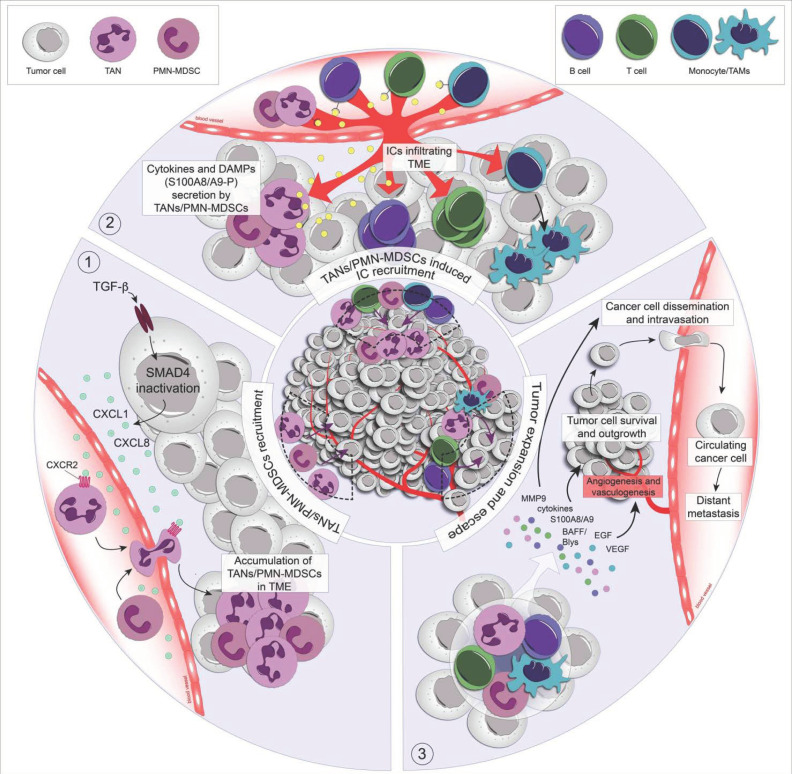
TAN recruitment promotes tumor growth and propagation. The interaction between TANs/PMN-MDSCs, tumor, and immune cells (ICs) is illustrated in the center. The details of various processes are highlighted at the periphery: (1) Recruitment of TANs/PMN-MDSCs to the tumor microenvironment (TME); TGF-β induces the inactivation of SMAD4 in cancer cells and the secretion of CXCL1 and CXCL8. These chemokines attract the circulating neutrophils to the TME via CXCR2. (2) Immune cell (IC) recruitment; once in the TME, neutrophils begin to secrete cytokines and DAMPs, which induce an adapted immune response and lead to IC recruitment. (3) Tumor cell growth and dissemination; TANs/PMN-MDSCs and recruited ICs secrete a plethora of mediators, which promote angiogenesis, tumor cell survival, and growth as well as intravasation.

A significant reduction of neutrophil infiltration has been associated with a decrease of inflammation-driven outgrowth of tumors and a more balanced immune surveillance [39], also seen in tumor-bearing animals where the depletion of neutrophils leads to an increase of anti-tumor effects of CD8^+^ T cells [40]. The CXCL/CXCR1/2 signaling axis is essential for the recruitment of neutrophils. In this regard, elegant studies carried out in mouse and zebrafish model systems highlighted the relationship between the recruitment of neutrophils and tumor progression through the blocking of CXCR1 and CXCR2 by gene knockout or using CXCR1/2 inhibitors [41]. It was reported that in the absence of CXCR2, neutrophils were selectively retained in the bone marrow [42]. An interesting recent finding stressed the link between SMAD4, a downstream mediator of TGF-β signaling, and neutrophil recruitment. The inactivation of SMAD4 in colorectal rectal cancer cells triggered an elevation of CXCL1 and CXCL8 expression, which in turn allowed an accumulation of neutrophils through their CXCR2 receptor engagement [43] (Figure 1). Given the critical role of CXCR1/2 in the recruitment of neutrophils to the tumor site, attempts were undertaken to block these receptors for cancer therapy. The molecule with the most promising benefits is a selective and reversible antagonist of CXCR1/2 named SX-682, which is currently in phase I clinical trials (Syntrix Biosystems Inc., clinicaltrials.gov identifier: *NCT03161431*). Consequently, CXCR1/2 are no longer activated by chemokines secreted by tumors, resulting in a decrease of neutrophil recruitment to the TME. Subsequently, this led to an inhibition of the inflammatory and immunosuppressive effects of neutrophils and was associated with a decrease in tumor growth and invasion [44]. Despite these encouraging results, one needs to keep in mind that the blockade of neutrophil recruitment could amplify the persistence of injury in the tumor microenvironment and, thus, foster the tumor progression in the long run.

Tumor-infiltrating neutrophils are a prominent source of cytokines and chemokines and can actively secrete hepatocellular growth factor (HGF) through the degranulation process [45]. These molecules act in a self-renewing autocrine/paracrine loop with a positive feedback, enabling the innate and adaptive immune system to influence the tumor development through the recruitment, activation, and polarization of other tumor-supportive neutrophils in the TME. Moreover, c-Met deletion in mouse neutrophils reduced their infiltration, underlining the fact that HGF-mediated Met activation promoted neutrophil trans-endothelial migration to the primary tumor and metastatic sites [46].

Neutrophils recruited to the tumor site are able to secrete high levels of cytokines and chemokines. Some of them, including CXCL1, CXCL8, CCL2, have been described as being involved in the establishment of an inflammatory environment, promoting the development of tumor whereas CCL17, TGF-β, IL-10, MCP-1/CCL2, MIP-1β/CCL4, and MIF attract tumor-associated macrophages and regulatory T cells that inhibit cytotoxic T and NK cells [47]. Besides cytokines and chemokines, VEGF and MMP-9 secreted by TANs are known to mediate the process of tumor angiogenesis [41] (Figure 1). TANs can also regulate B lymphocyte functions, including activation, antigen presentation, differentiation, proliferation, and immunoglobulin production by expression of “a proliferation-inducing ligand” (APRIL), and the B-cell activating factor/B lymphocyte stimulator (BAFF/BLyS) [47]. BAFF and APRIL are members of the TNF family and have been involved in the pathophysiology of blood cancer. The signal transduction pathways driven by BAFF and APRIL have not been fully elucidated, however, it has been shown that BAFF and APRIL constitute myeloma cell growth factors by activating pro-inflammatory signaling, such as NF-κB, MAP, and PI-3 kinase pathways, as well as by affecting the regulation of anti-apoptotic proteins (e.g., Bcl-2) [48].

## 3. The Ca^2+^ Signaling Pathway in TANs as a Promising Target for Cancer Therapy

Given the importance of the neutrophil cytokine signatures in the TME, cytokine secretion by TANs could represent an interesting control point to limit the propagation of tumors. Although the mechanisms of cytokine secretion by TANs remains largely elusive, some recent studies point out that Ca^2+^ signaling constitutes an important and common component of this process [49] with Ca^2+^ influx, having mostly pro-oncogenic impact [50,51,52]. In neutrophils, the store operated Ca^2+^ entry (SOCE) is considered the main mechanism responsible of Ca^2+^ mobilization. It involves a biphasic process including a release of intracellular Ca^2 +^ followed by an extracellular Ca^2+^ entry through the plasma membrane channels. Briefly, upon a pro-inflammatory stimulus, the activation of the plasma membrane receptors triggers phosphatidylinositol 4,5-bisphosphate hydrolysis, and *inositol* trisphosphate (IP3) generation, which results in the emptying of endoplasmic reticulum (ER) Ca^2+^ stores. The decrease of Ca^2+^ levels within the ER is sensed by the stromal interaction molecule (STIM) proteins. STIMs oligomerize and migrate towards the ER-plasma membrane junctions where they interact with the Orai Ca^2+^-selective ion channels, which are key players in Ca^2+^ homeostasis, and which can be induced by physiological receptor activation in cancer cells and immune cells. The STIM-Orai interaction then promotes Ca^2+^ influx into the cytosol (for more details see [53,54]), which regulates downstream signaling pathways involved in immune and metabolic functions of cells. In this context, the targeting of Orai and/or STIM to reduce oncogenicity could become a viable option in cancer therapy. An encouraging study provided evidence that inhibition of STIM in immune cells was able to block SOCE, resulting in decreased tumor progression [55]. Indeed, using conditional double knockout mice for T cell-specific Stim1 and Stim2 gene deficiency, the authors showed that both STIM1 and STIM2 are required for tumor killing functions of CD8^+^ T cells mediated by degranulation, expression of Fas ligand, TNF-α, IFN-γ production and exocytosis of cytolytic granules containing perforin. The suppression of such functions counteracted anti-tumor immunity by preventing the control of melanoma and colon carcinoma cell growth and their engraftment in STIM1- and STIM2- deficient cytotoxic T cells [55].

In the light of these results, it is tempting to speculate that the STIM homologs could also be targeted to regulate TAN functions and ultimately tumor progression. The intracellular Ca^2+^ signaling through STIM1/Orai1-mediated SOCE is required for modulating cytotoxic effector functions of neutrophils ranging from NADPH oxidase activation to granule exocytosis [56]. SOCE deficiency due to STIM gene mutations has been demonstrated to lead to defects in cytokine secretion [49]. Based on knockout mouse experiments, STIM2 appears to be a predominant isoform in the regulation of cytokine production. Indeed, during activation of the immune response by the insoluble β-1,3-glucan polysaccharide extracted from Saccharomyces cerevisiae, zymosan, or stimulation of Fcγ-receptors, TNF-α, IL-10, and IFN-γ levels in neutrophils were decreased in Stim2 and double Stim1/2 knockout, but not in Stim1 knockout mice [49,57]. Moreover, myeloid Stim1 deletion was not able to decrease chemokine/cytokine expression in an imiquimod-induced mouse psoriasis model, underlining that STIM1 was not essential for cytokine expression [58].

To date, the impact of the different neutrophil Orai isoforms on cytokine secretion has not been investigated. Therefore, it is not trivial to determine, which specific STIM and Orai isoforms to target because the blocking of specific isoforms dedicated to a specific function, such as the modulation of intracellular Ca^2+^ levels might aggravate unwanted effects on tumor progression. Indeed, it has previously been reported that drugs inhibiting SOCE affect anti-tumor immunity supported by CD8^+^ T, NK, and dendritic cells [59]. Thus, a total SOC channel inhibition could be responsible of an acceleration of cancer progression. In this context, Zhou et al. [59] reported that an optimal level of Ca^2+^ is required to obtain efficient cytotoxic effects of T and NK cells.

Intriguingly, a partial inhibition of Orai1 might result in an increase of perforin-dependent cancer cell killing by cytotoxic T cells [59], fueling the hypothesis that partial inhibition of Orai1-dependent SOCE may contribute to tumor elimination. Thus, the control of Ca^2+^ levels in TANs by modulating but not abolishing Ca^2+^ signaling through Orai/STIM could be an option to eventually promote the protective effects of neutrophils and to reduce their pro-oncogenic effects.

The distinct roles of STIM1 and STIM2 for neutrophil functions could be the key to more fine-tuned therapeutic interventions. As mentioned before, STIM1, in contrast to STIM2, has a very limited impact on cytokine secretion [49]. However, STIM1 has been shown to be involved in the control of ROS production [49,60,61]. Recently, reactive oxygen species (ROS) production has been proposed to mediate tumor cell apoptosis in breast cancer cell lines through an elevation of the intracellular Ca^2+^ concentration resulting from the activation of Ca^2+^-permeable TRPM2 channels [21]. The production of H_2_O_2_ by neutrophils in the TME might thus be responsible for the reduced capacity of circulating tumor cells to form metastases [21]. In this context, inhibition of STIM2 could lead to an inhibition of pro-oncogenic functions of TANs while neutrophil cytotoxicity mediated by STIM1 via the regulation of ROS production could remain. Along the same line, the role of different isoforms of Orai in cytokine secretion needs to be elucidated. The dysregulation of one of the Orai isoforms could abrogate the immunosuppressive effects of neutrophils in the TME by reducing inflammatory processes.

Another important aspect is that STIM and Orai isoforms might regulate differential cytokine secretion through the regulation of granule exocytosis. It has been postulated that pre-formed cytokines may be stored in neutrophil granules allowing for prompt release during exocytosis while de novo synthesized cytokines may only be liberated during constitutive exocytosis [62,63,64]. In this context, the intriguing question of whether STIM/Orai isoforms are able to selectively block the secretion of defined neutrophil granule contents remains to be addressed. Indeed, a selective inhibition of STIM/Orai isoforms could decrease MMP-9 secretion, a major secreted matrix metalloproteinase from neutrophils, which is involved in angiogenesis and tumor growth [65,66].

## 4. Targeting NETosis to Prevent Metastasis

**Figure 2:**
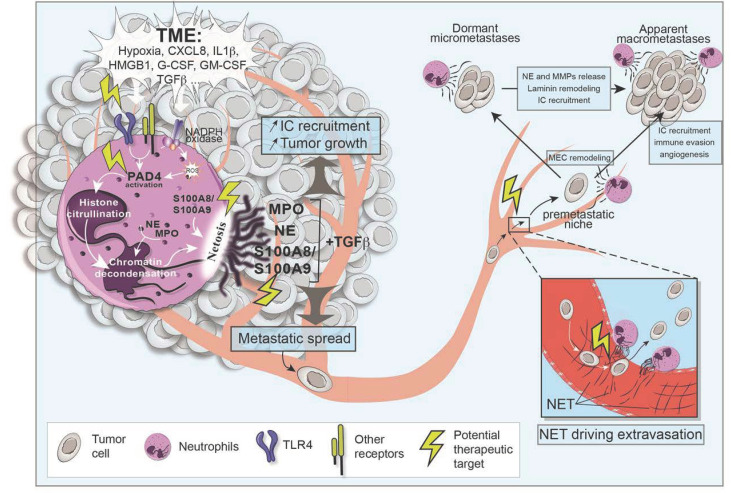
NETosis by TANs support metastatic dissemination and colonization at secondary sites. NET formation by TANs is favored by the tumor microenvironment (TME) and mediated by TLR4 signaling. It results in PAD4 activation, and MPO and neutrophil elastase (NE) translocation into the nucleus, which lead to chromatin decondensation and its release into the environment with cytosolic and granular proteins. The pro-tumorigenic effects of NETs occur through the action of potent proteases on matrix proteins and activation of inflammatory functions of immune cells (ICs). The adherence of NETs to the vessel wall facilitates metastatic disease progression by trapping circulating tumor cells and increases local vascular permeability. At the final stage of NETosis, a subset of molecules is released triggering an imbalance in the microenvironments and the emergence of metastatic niches. NETs can also promote cancer recurrence by activating dormant cancer cells through the remodeling of laminin and activation of integrin a3β1 signaling in resting cancer cells. Additionally, proteases associated with NETs can activate the complement and coagulation cascades leading to the recruitment of ICs and selectively degrade IL-6. The potential treatments that can block the NETosis process include the targeting of receptors (blockade of TLR4 or G-CSFR), PAD4 (BMS-P5 inhibitor), and DNA (inhibition of DNA de-condensation or treatment with DNase I-coated nanoparticles).

Besides the regulation of cytokine secretion by neutrophils, strategies to prevent the formation of neutrophil extracellular traps (NETs), a process specific to neutrophils [67], are promising to combat the spreading of cancer cells. NETosis is an antimicrobial mechanism intended to capture and destroy invading pathogens through the liberation of cytotoxic molecules. However, the release of inflammatory factors can promote the proliferation of tumor cells and inhibit their apoptosis [68]. The formation of NETs by TANs is favored by hypoxic conditions in the TME, characterized by the presence of pro-inflammatory cytokines, including IL-1β and IL-8 [69,70], growth factors (e.g., G-CSF) [71], intracellular mediators and high mobility group box 1 (HMGB1) (Figure 2), which has been reported to induce NETs through the TLR4 and p38 MAPK/ERK signaling pathways [72].

The pro-tumorigenic effects of NETs are based on their ability to promote metastasis. A subset of molecules is released at the final stage of NETosis, triggering an imbalance in the microenvironments and the emergence of metastatic niches. NETs release potent proteases, which can corrupt the immune response through degradation of matrix proteins and inhibition of immune cell functions [31,73], enabling metastatic cells to escape the immune response. In this context, neutrophil elastase (NE) can promote the production of IL-8, IL-1β, and TNF-α through the activation of several MMPs by macrophages. This process is dependent on Src kinase activation, highlighting the fact that NE also impacts integrins and integrin-mediated intracellular signaling [73].

NETs can furthermore promote cancer recurrence and metastasis by activating dormant cancer cells [31]. In a model of LPS- or tobacco smoke-induced lung inflammation in mice, it was postulated that NETs formed by neutrophils, mediate the proteolytic and sequential cleavage of laminin through the activation of NE and MMP9 [31]. The remodeling of laminin in turn activates integrin a3β1 signaling resulting in FAK/ERK/MLCK/YAP signaling in resting cancer cells and their subsequent awakening [31]. Moreover, the DNA mesh of NETs preferentially bound to laminin allowed proteases to more efficiently target their substrates.

Additionally, proteases associated to NETs can activate the complement and coagulation cascades, leading to the recruitment of immune cells and amplification of the immune response [74]. In a mouse model, spontaneous intestinal tumorigenesis was associated with an accumulation of neutrophils of a pro-tumorigenic N2 phenotype, which have a capacity to form NETs [75]. NETs allow the recruitment of components of the coagulation cascade through a scaffold formation triggering thrombus formation by induction of the fibrinogenesis. These events shift neutrophils towards a pro-tumorigenic (N2) phenotype, which undergo NETosis favoring tumor outgrowth [74,75] (Figure 2).

NETs can also facilitate metastatic disease progression by trapping circulating tumor cells. In a murine model of sepsis, NETs trap circulating lung carcinoma cells at the site of dissemination within the DNA mesh [64]. Rapid NET trapping was associated with the promotion of tumor cell adhesion to various distant target organs and thus the formation of metastasis [76,77,78]. Moreover, the adherence of NETs to the vessel wall can increase local vascular permeability since NETs retain their proteolytic activity permitting cancer cells to extravasate more easily [79].

Neutrophils show an increase ability to produce NETs in hematological malignancies such as chronic lymphocytic leukemia [80] and, therefore, could also participate in disease progression [81]. Moreover, in non-Hodgkin’s lymphoma, neutrophils have been reported to protect malignant B cells against the cytotoxic effect of therapies through interaction with lymphoma cells via the binding of CD11b/ICAM-1 expressed by neutrophils and B cells [82]. In multiple myeloma, neutrophils exhibit a reduction of their phagocytic ability and an immunosuppressive effect through arginase-1 [83]. Overall, the role of neutrophils has been far less studied in hematological cancer compared to solid tumors. In the future, more efforts need to be devoted to the understanding of mechanisms associated with neutrophil functions, and more particularly on the importance of NETs in the context of hematological malignancies, since neutrophils appear to be a preponderant actor in these diseases.

The prevention or the destruction of NETs offer a new basis for promising pharmacological interventions, bearing in mind not to hamper immune functions, and preserving the protective functions of neutrophils, including degranulation and phagocytosis, which are vital for the organism.

The conventional suicidal NETosis is a special form of programmed cell death known to require ROS production. Briefly, ROS triggers the translocation of neutrophil elastase into the nucleus where it degrades histones to promote nuclear de-condensation in synergy with myeloperoxidases. Peptidylarginine deaminase 4 (PAD4) also intervenes with the chromatin de-condensation by catalyzing the citrullination of histones. At the final stage of NETosis, after disruption of the nuclear and plasma membranes, chromatin is released into the environment with cytosolic and granular proteins, which are strongly bound to the highly decondensed chromatin (for more details on the process of classical NETosis, see [84,85,86]).

Based on our knowledge of the NETosis process, several options can be considered to inhibit the formation of NETs with the intention to prevent the formation of metastases (Figure 2).

(i) The inhibition of ROS production would result in the abortion of NETosis. In support of this, recent studies reported that genetic defects in the NADPH oxidase subunits reduced the pulmonary metastatic colonization after intravenous injection of tumor cells in mice [87]. The decrease of the formation of lung metastases was associated with the accumulation of activated anti-tumor T and NK cells [88,89]. However, although inhibition of ROS production is able to reduce NET formation and metastasis in mice, it is important to note that NADPH oxidase activity is essential for the killing of pathogens and for the prevention of secondary infections. Consequently, targeting NADPH oxidases in cancer therapy would likely not be an advisable approach. However, therapeutic interventions downstream of ROS production in order to maintain the ROS production while inhibiting NET formation could circumvent this problem. The molecules of the anthracycline class intercalate between base pairs of DNA and RNA strands during DNA or RNA synthesis [90] and disrupt the activity of topoisomerase 2, which is essential for DNA replication [91]. It has been reported that anthracyclines can suppress NETosis through their intercalating activity, resulting in the alteration of the de-condensation process and thus in the abrogation of NET formation [92].

(ii) Another strategy could be to prevent NET formation by targeting PAD4 and, thus, chromatin de-condensation. It has been demonstrated that a genetic downregulation of PAD4 decreased tumor growth and metastasis of colorectal cancer [93]. In addition, the pharmacological inhibition of PAD4 secreted by cancer cells prevents the citrullination of the metastatic liver extracellular matrix and subsequent mesenchymal-to-epithelial transition reducing metastatic growth [93]. Moreover, the repression of PAD4 activity by a novel pharmacological inhibitor BMS-P5 impairs citrullination of histone H3 and NET formation by neutrophils mediated by myeloma cells in humans and mice delaying disease progression [94]. A large panel of available PAD4 selective inhibitors have been developed in recent years. However, there is still a lack of data on the exact molecular mechanisms of PAD4 inhibitors and their effect on tumor progression and metastasis upon long-term treatments.

(iii) A feasible strategy to prevent pro-tumoral effects of NETosis would be to target the DNA mesh of NETs. DNase I has been shown to disrupt the extracellular DNA scaffold of NETs [95] in vivo during systemic administration [96]. In addition, NET inhibition by DNase-I impaired the invasion and migration of breast cancer cells in vitro and NET-digesting, DNase I-coated nanoparticles strongly reduced lung metastases of breast cancer in mice [97].

(iv) Next, the blocking of G-CSF by antibodies could also be an approach to reduce NET formation induced by tumor cells [71,98]. However, blocking G-CSF is difficult, as it is known to promote normal neutrophil functions and to prolong their survival [99]. In addition, G-CSF is essential for maintaining adequate numbers of circulating neutrophils by ensuring a normal neutrophil production during granulopoiesis and, thus, avoiding a pre-disposition to secondary infections [100]. Alternatively, the receptor for G-CSF (G-CSFR) could be targeted. A recent report by Wang et al. [101] underlined the ability of an anti-G-CSFR mAbs to reduce NETosis as well as pathogenic inflammation and injury in chronic conditions with no impact on bacterial and viral clearance [101]. Blocking the G-CSFR could thus represent a promising approach to reduce NETosis.

A blemish for the targeting of NETs in preventing tumor progression is that inhibition of NETs would likely not preserve the desirable anti-tumoral activity, which NETs might also have. For instance, MPO, present in NETs, has been reported to destroy melanoma cells and prevent their growth [102]. Another study showed that patients with chronic granulomatous disease have higher cancer incidences [103]. One of the explanations is that chronic granulomatous disease is associated with a defect of NADPH oxidase activity due to mutations in the NADPH gene [104]. The lack of superoxide production leads to a loss of formation of hydrogen peroxide, which constitutes the substrate of MPO.

Finally, the types of NETosis involved in the progression of metastasis are not clearly defined. The mechanisms characterizing the conventional suicidal NETosis are fundamentally different to vital NETosis. Vital NETosis occurs through the blebbing of the nuclear envelope resulting in maintaining the integrity of the plasma membrane and their normal functions [85]. Therefore, strategies to inhibit classical NETosis are not applicable to vital NETosis. A clear identification of the mechanistic differences linked of the type of NETosis occurring during tumor progression would be an important pre-requisite for developing efficient anti-cancer therapies targeting NETs.

## 5. Tumor Suppressive Properties of S100A8/A9 Proteins in PMN-MDSCs

Another therapeutic potential of targeting NETosis is connected to the ability of NETs to secrete the Ca^2+^-binding proteins S100A8 and S100A9 [105] (Figure 1), which are considered key players in linking inflammation and cancer [106]. Once secreted in the extracellular space, S100A8/A9 are able to recruit further neutrophils and tumor cells to inflammatory and metastatic sites, and to sustain inflammatory conditions, promoting tumor development, and creating a favorable environment for metastatic niche formation [107,108,109] (Table 2).

One of the main cell types that are attracted by S100A8/A9 secreted by neutrophils are myeloid-derived suppressor cells (MDSCs). MDSCs are defined as a heterogeneous population of myeloid cells [118,119] that constitute one of the crucial components of the TME. A major hallmark of MDSCs is their ability to support tumor progression through the inhibition of anti-tumor functions of T and NK cells [118,120]. In this sense, MDSCs play a critical role in tumor growth, angiogenesis, tissue invasion, and metastasis [121,122]. MDSCs are commonly divided into two major subpopulations, based on their morphological phenotypic characteristics: monocytic (M)-MDSCs and polymorphonuclear (PMN)-MDSCs [118,119,123]. PMN-MDSCs represent the most abundant population of MDSCs in most types of cancer and are able to infiltrate and accumulate in the TME where they co-exist with neutrophils and have been associated with poor prognosis [124,125,126]. PMN-MDSCs are not detectable in healthy individuals, but expand when tumors are present [127,128,129], where they facilitate tumor cell escape from immune surveillance [125,130,131,132].

PM-MDSCs accumulate both in solid tumors and in hematological malignancies in which they seem exert similar functions. In this sense, PM-MDSCs have been described in different types of hematological malignancies (e.g., multiple myeloma, non-Hodgkin lymphoma, chronic myeloid lymphoma, and myelodysplastic syndromes) to inhibit T cell surveillance, secrete suppressive IL-10 and TGF-β1, show an increase level of Arginase-1 and ROS production, as well as high levels of programmed death receptor ligand 1/programmed death receptor 1 [133,134,135,136,137,138].

A fundamental and still open question is whether PMN-MDSCs are causing tumor progression or whether they are recruited as a consequence of tumor progression [119].

PMN-MDSCs are considered to represent a subset of neutrophils with immunosuppressive functions that are generated and accumulated during tumor development [35,139,140]. It was reported that TAN1 possess the characteristics of classical neutrophils, whereas TAN2 have features of PMN-MDSCs [14] (Table 1). Recently, human PMN-MDSCs have been proposed to express lectin-type oxidized LDL receptor-1 (LOX-1) that can discriminate them from neutrophils [112]. The identification of such new marker could allow the delineation between neutrophils and PMN-MDSCs in peripheral blood and tumors of cancer patients.

The immunosuppressive functions of PMN-MDSCs make the control of this MDSC subset an appealing strategy for novel cancer immunotherapies. Moreover, it is clinically more realistic to specifically target a subgroup of functionally defined cells, such as PMN-MDSCs rather than the entire population of neutrophils.

Thus, one strategy could be based on blocking S100A8/A9 function in PMN-MDSCs. The excellent work of Sinha et al. [141] revealed that the total population of MDSCs from tumor-bearing mice possess receptors for S100A8/A9; these receptors have been identified as advanced glycation end products (RAGE). S100A8/A9 can activate the NF-κB pathway, which occurs through the carboxylated glycans-dependent binding of S100A8/A9. Carboxylated glycans are expressed on a subpopulation of *RAGE* found on cancer cells as well as on myeloid and endothelial cells [142,143]. They support cell proliferation via the activation of receptor-mediated signaling after S100A8/A9 binding [143]. In this context, in vivo blocking of interactions dependent on carboxylated glycan by the anti-carboxylated glycan antibody mAbGB3.1 reduced the incidence of colitis-associated colon cancer [142] and accumulation of MDSCs in the peripheral blood, spleen, bone marrow, and tumors from 4T1 tumor-bearing mice [141], but did not affect the immunosuppressive activity of MDSCs.

In addition, tumor infiltrating MDSCs have the ability to secrete S100A8/A9 [141], which creates an autocrine positive feedback loop allowing for their own accumulation resulting in enhanced survival of cancer cells. This process has been proposed to represent an important characteristic of pancreatic cancer progression [144,145,146]. Besides RAGE binding, S100A8/A9 can also interact with TLR4, which has been shown to lead to the promotion of lung pre-metastatic niches [108].

Noteworthy, no consensus has been reached to date on the receptor preferentially activated by S100A8/A9. The specificity of S100A8/A9 for a certain receptor is likely dependent on the cell type so that S100A8/A9 can affect different functions of target cells according to their pathological condition.

Using a murine Lewis lung carcinoma model, it has been demonstrated that the effects of S100A9 are probably tightly associated with the TGF-β pathway [117]. Indeed, S100A8 and S100A9 expression was significantly reduced in the presence of anti-TGF-β antibodies in tumor-bearing mouse sera. Inversely, S100A8 and S100A9 expression was increased in lungs cultured with TGF-β in the presence of VEGF-A and TNF-α [117]. Neutralizing anti-S100A8 and anti-S100A9 antibodies blocked the morphological changes and migration of CD11b/CD18 positive myeloid cells into the lungs of tumor-bearing mice through p38 signaling. Thus, S100A8 and S100A9, through the TGF-β axis, foster the development of metastasis in lung cancer; however, the specific pathways involved have not yet been elucidated. Even though an association between the S100A8-S100A9 and TGF-β signaling pathway has been established, it is not fully understood how S100A8 and S100A9 promote tumor development.

One possibility is that the TGF-β-mediated of S100A8 and S100A9 is dependent on mutations of SMAD4, a downstream mediator of TGF-β signaling [147]. Indeed, in different tumor types, inactivation of SMAD4 has been described to promote tumor progression by a number of mechanisms. Among them are inactivation of tumor suppressor genes APC, VEGF overexpression, increase of MMP-9 activity and of *GLUT1* levels, as well as recruitment of CCR1+ myeloid cells in colorectal cancer [148,149], activation of K-Ras mutations in pancreatic duct adenocarcinoma [150,151], inhibition of DNA repair mechanisms and finally, increased levels of genomic instability in lung and skin cancer [152,153].

In addition, based on data from colorectal and pancreatic adenocarcinoma cell lines, it has been shown that S100A9 is preponderant in the promotion of tumor growth in SMAD4-negative cancer cells [154] supporting a link between S100A9/TGF-β/SMAD4. However, further studies are needed to fill the knowledge gap on how the status of SMAD4 in tumoral cells influences the effects of S100A8 and S100A9 on the TME. Recently, S100A9 and S100A8 have been used to develop an attractive tool for the depletion of MDSCs in tumor-bearing mice. Quin et al. [155] generated peptide-Fc fusion proteins binding to MDSCs, which recognize native S100A9 and S100A8. The created “peptibody” was able to deplete MDSCs systemically (in the peripheral blood and spleen) and intratumorally, resulting in inhibition of tumor growth in a lymphoma mouse model. Interestingly, treatment with these peptibodies did not affect pro-inflammatory immune cell types (e.g., dendritic cells) or myeloid precursor cells resident in the bone marrow affected [155]. Taken together, the blocking of S100A8 and S100A9 secretion could be an attractive way to decrease the stimulatory impact of neutrophils on the development of tumors.

## 6. Conclusions

Although TANs are now considered potential therapeutic targets, especially in the context of improving or complementing existing immunotherapies for treatment of various cancers, we still have a long road ahead before we can fully understand and modulate specific TAN functions in cancer patients. Most of the studies reported in this review have been derived from mouse models and TAN1 and TAN2 phenotypes have only been defined in mice [156,157]. Most of the available data on neutrophil functions in humans have been obtained from circulating neutrophils in patients with cancer rather than from healthy individuals. Consequently, the extrapolation of these results to “normal” neutrophils or TAN functions within specific tumor tissues should be made with caution. If the pro- and anti-tumorigenic role of TAN2 and TAN1, respectively, can be confirmed in humans, the potential switching of TAN2 into TAN1 could become a worthwhile strategy in cancer therapy. Alternatively, if TAN2 cells turn out to be PMN-MDSCs, strategies to neutralize the immunosuppressive functions of PMN-MDSCs could apply (for review [158]). Only the identification of markers could favor one scenario over another, namely if TAN1 are in fact bona fide neutrophils, and TAN2 related to PMN-MDSCs, or TAN1 and TAN2, correspond to two different phenotypes of neutrophils.

In this context, entinostat, a selective and oral class I histone deacetylase inhibitor, provided encouraging results in murine models for solid tumors [159]. In combination with immune checkpoint inhibitors, such as PD-1/PD-L1 antagonists, entinostat enhanced the anti-tumoral effects of PD-1 inhibition in two syngeneic mouse models for lung and renal cell carcinoma [159]. This combinatorial treatment also upregulated anti-tumor cytokine/chemokine release in vivo and, thus, altered the immunosuppressive TME resulting in increased anti-tumor effects of anti-PD-1 and prolonged survival [159]. Entinostat was shown to downregulate COX-2 and ARG-1 expression, and reactive oxygen species (ROS) production, and inhibit the activation of the transcription factor STAT3 in PMN-MDSCs, which lost their immunosuppressive activity [159].

Clinical trials are currently ongoing with entinostat alone (SNDX-275 in phase 2) or combined with anti-PD-1 (entinostat + pembrolizumab in phase 2), for refractory Hodgkin’s lymphoma (Syndax Pharmaceuticals, clinicaltrials.gov identifier: *NCT00866333*). The modulation of TAN functions in the microenvironment of solid tumors should, however, not disturb other important functions in order to prevent detrimental side effects. Nevertheless, the potential benefits of blocking pro-tumorigenic TAN properties are still an appealing and worthwhile strategy to explore for future cancer treatments. Alternatively, an emerging and promising concept for cancer therapy based on the neutrophil properties, would entail the delivery of therapeutics at the tumor site by viable neutrophils or neutrophil membrane-derived nanovesicles, since the infiltration of these cells is associated with tumorigenesis. Such technologies are in their early stages and will have to await further data to prove their therapeutic potential [158,160,161].

## Figures and Tables

**Table 1:** Functional properties of TAN1 and TAN2.

TAN1 = “Classical Neutrophils”? (Anti-Tumoral)			TAN2 = PMN-MDSCs? (Pro-Tumoral)		
Mature/Segmented Nucleus/High-Density Neutrophils			Immature/Ring-Shaped Nucleus/Low-Density Neutrophils		
CD66b^+^/CD11b^+^/CD14^−^/HLA-DR^+^/CD177^+^/CD15^hig^			CD15^+^/HLA-DR/CD11b^+^/CD14^−^/CD33^+^/Lox-1^+^		
Role	Mode of Action	Tumoral Effect	Role	Mode of Action	Tumoral Effect
ROS production	• TRPM2 activation → lethal Ca^2+^ entry [21]	Tumor growth inhibition	ROS production	• DNA mutations [22]	Tumor promotion /progression
Chemokine/cytokine secretion	• Leukocyte recruitment • Proliferation of T-cells [23]	Immune anti-tumor response	Chemokine/cytokine secretion	• ↑ CCL17 expression secretion → T_regs_ recruitment to the TME [23]	Tumor progression
Fas signaling	• Activation of caspase cascade [24]	Apoptosis of cancer cells	Neutrophil elastase secretion	• Activation of EGFR, TLR4→ ERK-dependent gene transcription [25] • Degradation of insulin receptor substrate 1 → PI3K-Akt activation [26] • Inactivation of thrombospondin-1 • Cleavage of EMILIN1 [27]	Tumor proliferation
MMP-8 release	• ↓ β1-integrin activity [28] • Cleavage of cytokines [29] • Cleavage of decorin → ↓ active TGF-β → ↓ miR-21 expression → ↓ PDCD4 [30]	Tumor suppression	NET formation	• TME remodeling [31] • Activation of dormant cancer cells [31]	Metastasis
			MMP-9 and VEGF secretion	• Remodeling of ECM membrane → TGF-β activation [32,33] • ↑ vascular permeability [32,33] • ↑ endothelial cell growth [32,33]	Tumor angiogenesis
			Arginase secretion	• ↓ cytotoxic CD8^+^T cell effects [34]	Immuno-suppression

**Table 2:** Role of S100A8/S100A9 in the tumorigenesis.

Apoptotic/cytotoxic effects	• Cleavage of pro-caspase by zinc sequestration	[110]
	• Pertubation of the mitochondrial pathway	[111]
	- Absence of cytochrome *c* release	[111]
	- Induction of caspase activity	[111]
	- Alteration of the mitochondrial membrane potential	
Cell proliferation	• Recruitment of MDSCs	[112]
	• Inhibition of dendritic cell differentiation	[113]
	• MAPK phosphorylation and NF-κB activation via RAGE	[114]
Cell differentiation	• Increase of NF-κB activation by epithelial NADPH oxidases	[115]
	• Increase of involucrin and filaggrin expression	[116]
Adhesion and invasion	• Attraction of Mac-1+ myeloid cells	[117]

## Abbreviations

HGF: Hepatocyte growth factor; NE, neutrophil elastase; NET, neutrophil extracellular trap; PAD4, peptidylarginine deiminase 4; PMN-MDSC, polymorphonuclear myeloid-derived suppressor cell; ROS, reactive oxygen species; STIM, stromal interaction molecule; SOCE, store-operated Ca^2+^ entry; TAN, tumor-associated neutrophil; TME, tumor microenvironment.
